# Increased Free Radical Generation during the Interaction of a Quinone-Quinoline Chelator with Metal Ions and the Enhancing Effect of Light

**DOI:** 10.3390/ph16081116

**Published:** 2023-08-08

**Authors:** Olga Yu. Selyutina, Simon V. Babenko, Irina A. Slepneva, Nikolay E. Polyakov, George J. Kontoghiorghes

**Affiliations:** 1 Institute of Chemical Kinetics & Combustion, Novosibirsk 630090, Russia; olga.gluschenko@gmail.com (O.Y.S.); s.babenko@tomo.nsc.ru (S.V.B.); slepneva@kinetics.nsc.ru (I.A.S.); polyakov@kinetics.nsc.ru (N.E.P.); 2 International Tomography Center, Novosibirsk 630090, Russia; 3 Postgraduate Research Institute of Science, Technology, Environment and Medicine, Limassol CY-3021, Cyprus

**Keywords:** anthraquinones, quinoline, metal ions, iron, copper, anticancer activity, free radicals, toxicity, EPR, NMR, CIDNP, light, photodynamic therapy

## Abstract

Schiff bases and similar molecules forming metal complexes may cause redox effects, which may also be influenced by light. Anthraquinones such as doxorubicin and idarubicin are widely used antitumor agents, which can generate reactive oxygen species (ROS), stimulated by both the presence of iron and copper ions and also by light. The generated ROS can cause DNA scission, cell membrane oxidation, and many other toxic effects. The redox activity of the quinone-quinoline chelator 2-phenyl-4-(butylamino)naphtho [2,3-h]quinoline-7,12-dione (Q1) was investigated in the presence of iron, copper, and zinc. The influence of light in these interactions was also examined. The chemically induced dynamic nuclear polarization (CIDNP), nuclear magnetic resonance (NMR), and electron paramagnetic resonance (EPR) methods were used to elucidate the molecular changes and ROS generation effects of the Q1 metal interactions. A model electron transfer reaction system between 1,4-dihydropyridine and Q1 was utilized to demonstrate that the chelate complexes of Q1 with both Fe(III) and Cu(II) ions were more redox active than Q1 itself. Similarly, CIDNP and NMR data showed that the concentration dependence of the free radicals yield is much higher in the presence of Fe(III) and Cu(II) ions, in comparison to Zn(II), and also that it increased in the presence of light. These findings underline the role of transition metal ions and Q1 in cyclic redox chain reactions and increase the prospect of the development of copper- and iron-based chelating agents, including Q1 and its derivatives, for anticancer therapy. Furthermore, these findings also signify the effect of light on enhancing ROS formation by Q1 and the prospect of utilizing such information for designing target specific anticancer drugs for photodynamic therapy.

## 1. Introduction

Cancer is one of the major diseases with huge impact on the whole of humanity, with high morbidity and mortality rates. In the year 2020, there were 19.3 million new cancer cases and about 10 million related deaths worldwide [1]. The design of new anticancer drugs is an important and imminent task for many scientists around the world.

Many cytotoxic drugs, including the anthracyclines doxorubicin and idarubicin, are widely used for the treatment of cancer. Besides the primary mechanism of action of these anthracyclines, which is considered to be DNA intercalation [2,3,4,5,6], different studies have indicated that another mechanism of their activity is related to the generation of reactive oxygen species (ROS) [6,7,8,9,10,11,12]. Thus, it has been shown that the ROS generated by the anthraquinone part of the molecule could destroy cellular membranes by stimulating lipid peroxidation [2,3,4]. 

**Scheme 1:**
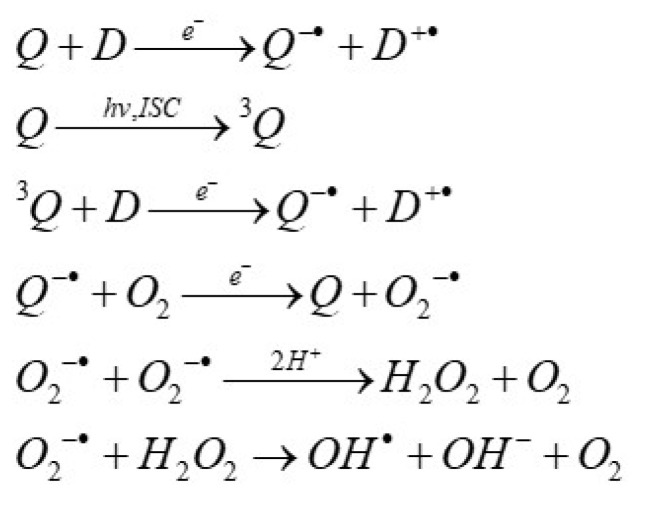
A list of reactions describing the quinone (*Q*)-induced reactive oxygen species generation in the presence of electron donors (*D*) in dark and light conditions. ^3^*Q* is quinone triplet state, ***h****v* indicates the photoexcitation, and ISC is intersystem crossing.

It is also known that the above and other anthracyclines could influence mitochondrial electron transport [13,14,15]. Furthermore, they can produce ROS in the dark as a result of reduction by such agents such as ascorbic acid, nicotinamide adenine dinucleotide (NADH), and reduced glutathione (GSH), or they can act as photosensitizers [5,10,16,17,18], as shown in the reactions in Scheme 1:

In the presence of oxygen, a semiquinone radical may be oxidized, forming superoxide ion (O_2_^−^·), which reacts fast with another O_2_^−·^ radical, causing H_2_O_2_ formation. H_2_O_2_ can react directly with Fe^2+^ to produce toxic hydroxyl (•OH) radicals via the Fenton reaction [4]. The reactions described by Scheme 1 take place for quinones with appropriate redox potentials.

Several anthraquinones are also able to produce ROS and react with peptides and DNA, as well as cause cytotoxic effects in the presence of light [10,16,19,20,21,22]. For example, mitoxantrone demonstrates a significant increase in cytotoxic activity under light exposure on MCF-7 cells, whereas emodin and aloe-emodin can be used as photosensitizers for other cancer cell lines [21,23]. Under certain conditions, anthraquinones can be used as photosensitizer agents for photodynamic therapy, a procedure involving the injection of a photosensitizer followed by irradiation, which results in the excitation of the photosensitizer and increased production of ROS that cause cytotoxicity in tumor cells [22]. 

The ability of anthraquinones to form chelate metal complexes can also influence their anticancer activity and toxicity [24,25,26,27,28,29]. There is a significant difference in the efficiency of the DNA damage by each of the anthraquinones and their metal complexes. For example, a decrease in DNA damage is observed in the case of alizarin and emodin complexes with Mn(II). In contrast, emodin complexes with Cu(II) are redox active and involved in the Fenton reaction, generating hydroxyl (•OH) radicals, which, in turn, enhance the modification of nucleobases [25,30]. 

**Figure 1:**
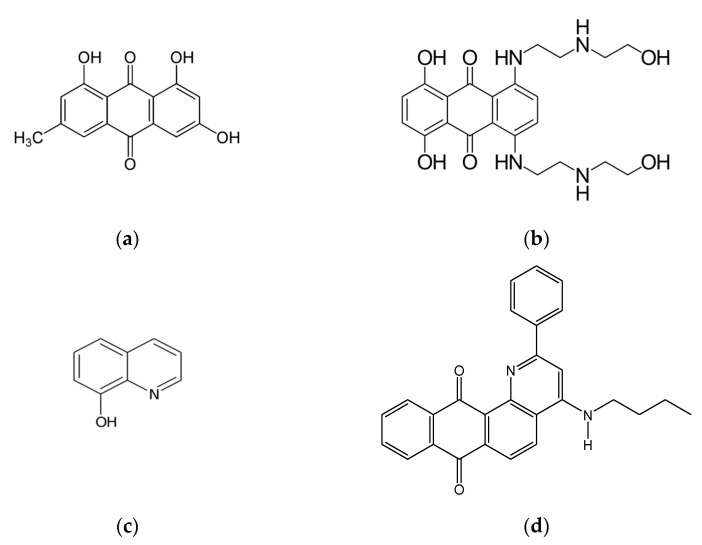
The chemical structures of some anthraquinones and quinolines with anticancer activity: emodin (**a**), mitoxantrone (**b**), 8-hydroxyquinoline (**c**), and the anthraquinone/quinoline 2-phenyl- 4-(butylamino)naphtho [2,3-h]quinoline- 7,12-dione (Q1) studied in this work (**d**).

It is also interesting that the quinone-quinoline chelator 2-phenyl- 4-(butylamino) naphtho [2,3-h]quinoline-7,12-dione (Q1, Figure 1) used in the present study exhibited higher activity in terms of ROS production than doxorubicin [5]. The chelating center of Q1 comprises the nitrogen of the quinoline part, which acts as a Schiff base, and the adjacent oxygen of the quinone. Quinoline and its derivatives have wide biological and pharmacological applications, including anticancer and antimicrobial activity [31,32,33,34,35,36,37,38,39,40]. Similar diverse activity has also been shown by transition and other metal complexes of quinoline and its derivatives, such as 8-hydroxyquinoline (Figure 1) [41,42,43,44,45,46,47,48,49,50]. In contrast, quinones with redox properties similar to Q1, but without a chelating center, have shown much less cytotoxic activity toward cancer cells [5]. This difference in activity seems to stem from different factors, including the change in the reduction potential of quinone upon metal complex formation [17]. 

The essential transition metals, iron and copper, play a central role in the redox reactions in biological systems, including the enhancement of ROS production, enzymatic catalysis, electron transfer, and drug metabolism [51,52]. Their redox activity is also influenced by natural and synthetic chelators and chelating drugs [53,54,55,56,57].

There are many other chelator properties and parameters influencing free radical generation and the cytotoxic activity of chelators and their metal complexes. The original concept and findings of the increased cytotoxicity of metal complexes against cancer cells was reported about 35 years ago, where the lipophilic iron complexes of 8-hydroxyquinoline, omadine, and tropolone were shown to exhibit much higher anticancer activity than doxorubicin and the chelators themselves [58,59]. Similar findings were reported recently with the lipophilic iron and copper complexes of thiosemicarbazones, which are also under development as anticancer drugs [60].

Recently, an enhancement in the production of short-lived radicals under light irradiation has also been demonstrated in vitro by Zn^2+^ quinone complexes [17]. In this context, the stability and stoichiometry of the chelates of Q1 with other metal ions were also studied, for which direct evidence of chelate formation and ROS production enhancement had previously been observed [17,61]. Based on these data, the formation of short-lived radical species in the reactions involving chelates composed of Q1 and Cu^2+^ or Fe^3+^ ions, both in dark and light conditions, were studied.

**Scheme 2:**
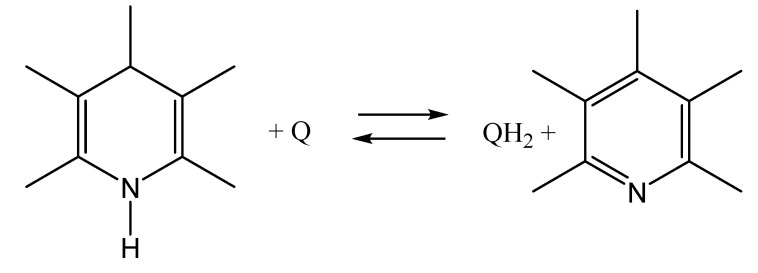
The oxidation/reduction reactions between 1,4-dihydropyridine (**left**) and pyridine (**right**), where Q is quinone and QH_2_ is hydroquinone.

The synthetic derivatives of 1,4-dihydropyridine (Scheme 2) are usually employed to model the action of the coenzyme NADH [62]. The action of natural NADH coenzymes is based on the conversion of NADH into NAD+, which is realized due to the oxidation of 1,4-dihydropyridine (left structure in Scheme 2) to pyridine (right structure in Scheme 2), or the reverse reaction of the reduction of pyridine to 1,4-dihydropyridine [62]. In order to study this photo reaction, the chemically induced dynamic nuclear polarization (CIDNP) technique has been used, which is an indirect experimental method for studying free radicals’ fate in complex chemical and biochemical processes [63,64,65,66,67,68,69,70]. 

There are thousands of promising anticancer compounds and metal complexes that have been reported in the literature, where ROS generation may enhance anticancer activity [71]. Similar mechanisms and modes of action are also investigated in the present study, with ultimate aim the design of target-specific anticancer drugs for photodynamic therapy. 

## 2. Results and Discussion

### 2.1. Formation of the Semiquinone Radical in Dark Conditions in the Presence of Reducing Agents

The redox effects of Q1 and its metal complexes were studied using a model reaction system involving the photo-oxidation reaction of 2,6-dimethyl-3,5-dicarbmethoxy-1,4-dihydropyridine (DHP), as shown in Scheme 2. 

**Figure 2:**
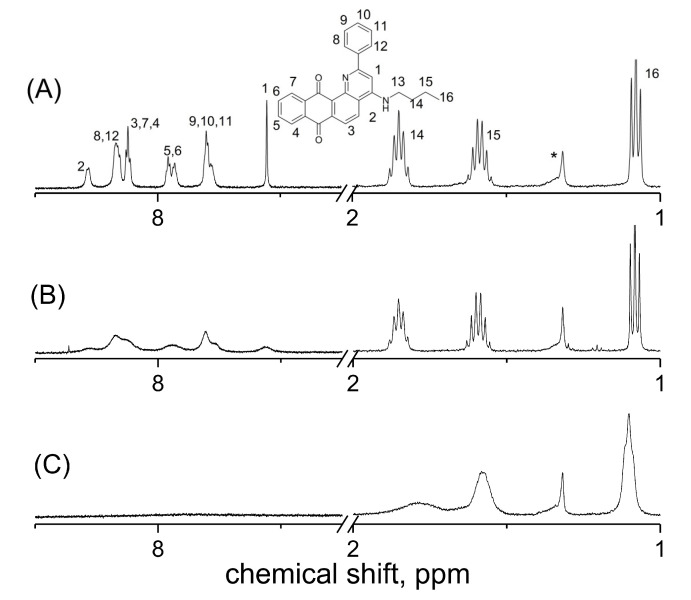
^1^H NMR spectra of 2 mM 2-phenyl-4-(butylamino)naphtho [2,3-h]quinoline-7,12-dione (Q1). (**A**) In the presence of Q1 in deuterated methanol (CD_3_OD), (**B**) in the presence of Q1 plus 2,6-dimethyl-3,5-dicarbmethoxy-1,4-dihydropyridine in CD_3_OD, and (**C**) in the presence of Q1 plus ascorbic acid in CD_3_OD. The numbers on the spectrum correspond to numbers of protons in the molecular structure of Q1 given above. (*) corresponds to the solvent impurity.

The behavior of Q1 and DHP in the absence/presence of metal ions in dark conditions was studied prior to exploring the photochemical reactions. Figure 2 represents the ^1^H NMR spectra of Q1 in the absence and presence of DHP, in deuterated methanol (CD_3_OD or MeOD). 

**Figure 3:**
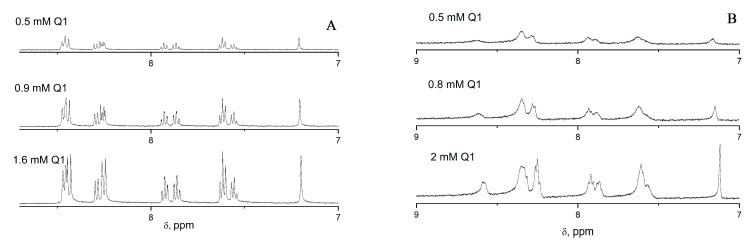
^1^H NMR spectra demonstrating the dependence of the line width of the aromatic protons of 2-phenyl-4-(butylamino)naphtho [2,3-h]quinoline-7,12-dione (Q1) on the concentration of Q1 inacetonitrile-d_3_ (ACN-d_3_) (**A**) and in deuterated methanol (CD_3_OD) (**B**).

A significant line broadening of the signals of the aromatic protons in the stationary ^1^H NMR spectrum in the presence of DHP was observed. Moreover, an even more significant line broadening was observed in the presence of ascorbic acid, which is a stronger reducing agent. It appears that such a strong line broadening may be caused by the electron exchange between the quinone and its paramagnetic derivative—semiquinone. The capability to observe this effect in the stationary NMR spectrum indicates that the radical is long-lived. The broadening of the Q1 aromatic lines in the ^1^H NMR spectra was observed even in the absence of a reducing agent, which was depended on the solvent used. Though at the Q1 concentration used (~1 mM), no evidence of semiquinone radicals in MeOD was observed in the EPR spectrum, the NMR spectrum of the aromatic lines of Q1 was apparently broadened compared to the aliphatic lines and aromatic lines of Q1 in acetonitrile-d_3_ (ACN-d_3_) (Figure 3).

Moreover, the concentration dependence of Q1 showed no influence on the line width; therefore, this suggests that the electron did not come from another Q1 molecule, but was more likely to come from the solvent—methanol. This is in agreement with previously reported information suggesting that a semiquinone radical anion was observed in the presence of ethanol for another anthraquinone derivative, namely anthraquinone-disulfonate [72].

**Figure 4:**
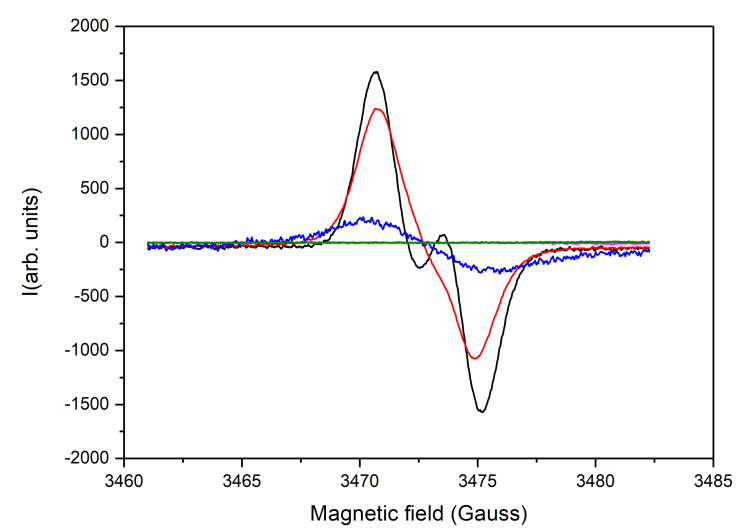
Steady-state EPR spectra of 1 mM 2-phenyl-4-(butylamino)naphtho [2,3-h]quinoline-7,12-dione (Q1) plus 7 mM ascorbic acid (black); 1 mM Q1, plus 7 mM ascorbic acid, plus 1 mM glutathione (red); 1 mM Q1, plus 5 mM 2,6-dimethyl-3,5-dicarbmethoxy-1,4-dihydropyridine (DHP) (blue); 1 mM Q1, plus 5 mM DHP plus 2 mM CuSO_4_ (green); and 1 mM Q1 plus 5 mM DHP plus HCl (purple); All solutions are aerobic.

An EPR spectrum was obtained under the conditions used in the present work, i.e., with methanol as a solvent and DHP as a reducing agent (Figure 4). It should be noted that the semiquinone radical anion has also been previously identified by EPR in a different solvent with different reducing agents [5].

A strong EPR signal is observed in the presence of DHP and ascorbic acid, which, in terms of structure and position (g-factor), resembles that of the semiquinone radical anion [5]. Both the DHP and ascorbic acid lead to the formation of the semiquinone radical anion, however, in the presence of ascorbic acid, the characteristic doublet of the ascorbate radical is also observed, which disappears upon the addition of glutathione (Figure 3A). In addition, the yield of the semiquinone is apparently much higher in the case of ascorbic acid, which also correlates with the NMR spectra presented in Figure 2. 

It can therefore be suggested that the observed line broadening in the NMR spectrum (Figure 2) can be attributed to the chemical exchange between the paramagnetic semiquinone radical (Q1^●−^) and its diamagnetic predecessor, Q1:

$$Q1^{●-}+Q1\;\underset{}{↔}Q1+Q1^{●-}$$

**Figure 5:**
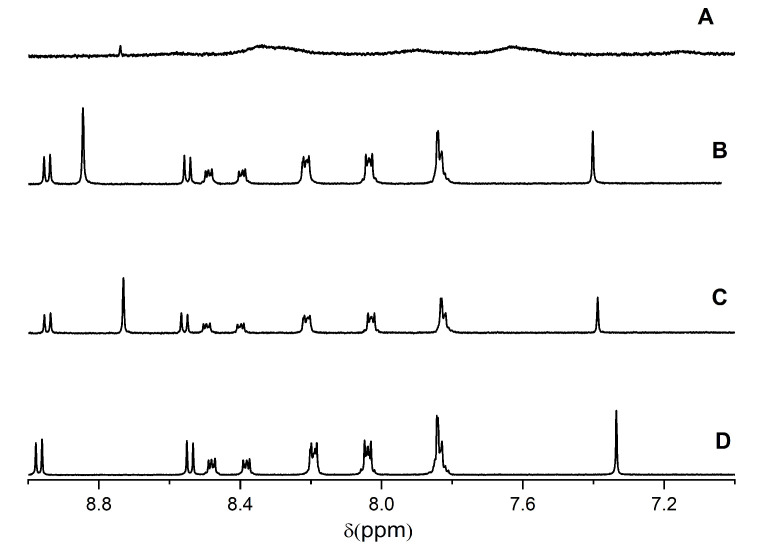
^1^H NMR spectrum of (**A**) 1 mM 2-phenyl- 4-(butylamino)naphtho [2,3-h]quinoline-7,12-dione (Q1) plus 3 mM 2,6-dimethyl-3,5-dicarbmethoxy-1,4-dihydropyridine (DHP) in CD_3_OD, (**B**) 0.7 mM Q1 plus 2.2 mM DHP plus 1.8 mM Cu^2+^, (**C**) 1 mM Q1 plus 3 mM DHP plus 3 mM Fe^3+^, and (**D**) 2 mM Q1 plus DCl in CD_3_OD (10% D_2_O).

Upon the addition of Cu^2+^ to the sample containing Q1 and DHP, the line corresponding to the semiquinone radical anion disappears, which is probably related to the scavenging of the radicals by Cu^2+^ (Figure 4, green line). No semiquinone radical is also observed if strong acid is added (Figure 4, purple line). These findings suggest the protonation of the semiquinone anion radical with the formation of Q1H^●^, which could have a much shorter lifetime than its deprotonated derivative [73]. In fact, the results obtained with the steady-state EPR can again be correlated with those obtained with the ^1^H NMR (Figure 5).

In the presence of metal ions (e.g., Cu^2+^) or H+, the ^1^H NMR spectrum of the quinone changes significantly, and these changes are related not only to the shift of the Q1 NMR line due to the formation of the metal complex, but the NMR lines also narrow, which indicates that the chemical exchange between Q1 and a semiquinone radical is inhibited.

### 2.2. Formation of the Semiquinone Radical in the Presence of Light 

The photoinduced reaction between quinones and reducing agents, such as NADH and its synthetic analogues, 1,4-DHPs, was previously studied using the CIDNP technique [17,62]. An increase in the CIDNP intensity of the protons of DHP and the corresponding pyridine product (Pyr) was also observed in the photoreaction between Q1 and DHP in the presence of Zn^2+^ [17]. 

The Gibbs energy of the photochemical electron transfer reaction can be described by the Rehm–Weller equation:

ΔG_et_ = E^1/2^(ox) − E^1/2^(red) − E_T_ + ΔE_coup_, (1)

where E^1/2^(ox) and E^1/2^(red) are the oxidation and reduction potentials, respectively, E_T_ is the energy of the photoexcited state, and ΔE_coup_ is the term related to the Coulomb coupling of the charged radicals. 

**Figure 6:**
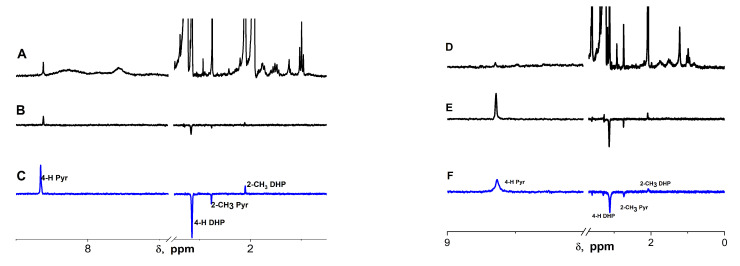
^1^H NMR and ^1^H CIDNP spectra of the photoreaction products of 2-phenyl-4-(butylamino)naphtho [2,3-h]quinoline- 7,12-dione (Q1) and 2,6-dimethyl-3,5-dicarbmethoxy-1,4-dihydropyridine (DHP). (**A**) ^1^H NMR of Q1 and DHP in 1:1 acetonitrile-d_3_:MeOD, (**B**) ^1^H CIDNP of 2 mM Q1 with 10 mM DHP in 1:1 acetonitrile-d_3_:CD_3_OD, and (**C**) ^1^H CIDNP of 2 mM Q1 with 10 mM DHP with 2 mM CuSO_4_ in 1:1 acetonitrile-d_3_: CD_3_OD. (**D**) ^1^H NMR of Q1 and DHP in CD_3_OD, (**E**) ^1^H CIDNP of 2 mM Q1 and 8.5 mM DHP in CD_3_OD, and (**F**) ^1^H CIDNP of 2 mM Q1 and 8.5 mM DHP in CD_3_OD + 2 mM FeBr_3_.

In the present work, the chelate complexes of Q1 with Cu^2+^ and Fe^3+^ ions were tested, showing an enhancement of the CIDNP intensity, which was well seen in the case of Cu^2+^, but although the enhancement still existed for Fe^3+^, this did not manifest itself under the conditions of the study, for the reasons discussed below (Figure 6).

**Scheme 3:**
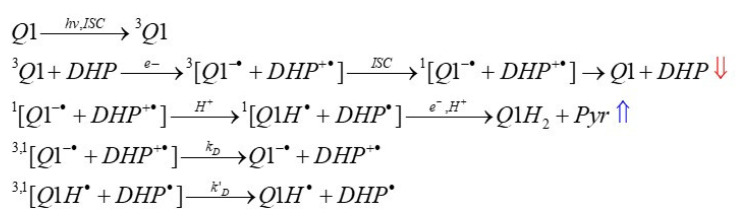
The reaction scheme of photoinduced interaction of the quinoline-quinone 2-phenyl-4-(butylamino)naphtho [2,3-h]quinoline-7,12-dione (Q1) with 2,6-dimethyl-3,5-dicarbmethoxy-1,4-dihydropyridine (DHP). Here, the radical species in brackets represent a radical pair (RP). The red and blue arrows indicate the observed polarization of the 4-H of DHP and pyridine, respectively. The detailed structures of DHP radicals and their magnetic resonance parameters have been previously described [62].

Considering the data on the DHP photo-oxidation presented in the literature [62], the reaction between Q1 and DHP can be described as shown in Scheme 3:

In general, the polarization of the DHP and Pyr protons was observed only due to the low values of the hyperfine constants on the Q1 protons and the broadening effects in the ^1^H NMR spectrum demonstrated above. 

**Figure 7:**
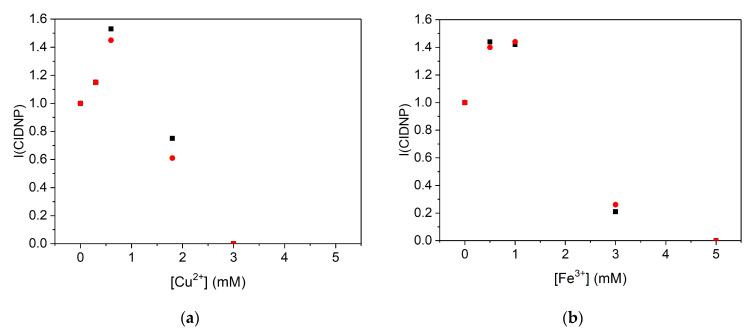
The effect of iron and copper on the CIDNP intensity (I(CIDNP)) during the photoreaction of 2-phenyl- 4-(butylamino)naphtho [2,3-h]quinoline- 7,12-dione (Q1) and 2,6-dimethyl-3,5-dicarbmethoxy-1,4-dihydropyridine (DHP). (**a**) Dependence of CIDNP intensity of H-4 of pyridine (▪) and DHP (●) on the concentration of Cu^2+^ in the photoreaction between Q1 (0.7 mM) and DHP (2.2 mM). (**b**) Dependence of CIDNP intensity of H-4 of pyridine (▪) and DHP (●)on the concentration of Fe^3+^ in the photoreaction between Q1 (1 mM) and DHP (3 mM).

The dependence of the CIDNP intensity on the concentration of CuSO_4_ and FeBr_3_ appeared to be different from what was observed for Zn^2+^ and Ca^2+^, where first, an increase in the CIDNP intensity was observed until reaching plateau corresponding to the stoichiometric ratio of the Q1 and Zn^2+^ (or Ca^2+^) complex (Figure 7) [16]. In those studies, further increases in the Zn^2+^ or Ca^2+^ concentration did not lead to a decrease in the CIDNP intensity. In contrast, in the case of Cu^2+^ and Fe^3+^, no plateau was observed, but a decrease in the CIDNP intensity was as the metal ion concentration increased. The reason for this was that Cu^2+^ and Fe^3+^ are excellent oxidizing agents and are capable of oxidizing the DHP. Another reason for the CIDNP decay at a high metal ion concentration might have been the electron transfer from the semiquinone radical to the metal ion [5]. Though it is difficult to obtain accurate CIDNP enhancement factors for the complexes of Q1 with Cu^2+^ or Fe^3+^ from these graphs, they nevertheless provide qualitative evidence of enhancement, which can be taken as a low margin.

As previously suggested, the CIDNP intensity was proportional to the concentration of the radical pairs ($I_{CIDNP}∼\left[RP\right]\cdot n$), where [*RP*] is the concentration of the radical pairs and n is the amplification factor per one radical pair, which depends on the magnetic resonance parameters [65]. Considering that no significant change in the magnetic resonance parameters occurred upon complex formation, since the unpaired electron was still localized on the quinone, the increase in the CIDNP intensity (I_CIDNP_) observed in Figure 7 could only be due to increase in [*RP*], which resulted from the increase in the ^3^Q1 quenching rate constant (k_q_). Furthermore, one can also notice from Figure 7 that, at a high concentration of Cu^2+^ and Fe^3+^ ions, the observed CIDNP intensity decreased significantly.

**Figure 8:**
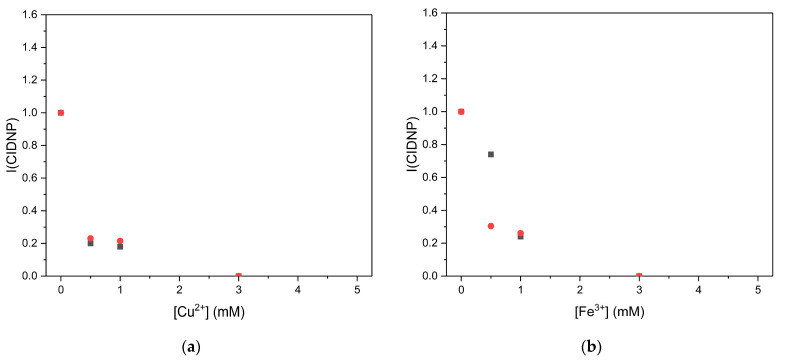
The effect of iron and copper on the CIDNP intensity (I(CIDNP)) during the photoreaction of emodin and 2,6-dimethyl-3,5-dicarbmethoxy-1,4-dihydropyridine (DHP). (**a**) Dependence of CIDNP intensity of H-4 of pyridine (▪) and DHP (●) on the concentration of Cu^2+^ in the photoreaction between emodin (1 mM) and DHP (3 mM). (**b**) Dependence of CIDNP intensity of H-4 of pyridine (▪) and DHP (●) on the concentration of Fe^3+^ in the photoreaction between emodin (1 mM) and DHP (3 mM).

To compare the activity of Q1 with known drugs, the photoreactions between emodin and DHP and mitoxantrone and DHP were also studied. Both demonstrated an increase in cytotoxic activity under light conditions in different cell lines [21,23]. For mitoxantrone, no CIDNP effects on DHP or pyridine protons were observed, both in the absence and in the presence of metal ions. For emodin, the dependence of the CIDNP intensity on the concentrations of CuSO_4_ and FeBr_3_ appeared to be different from what was observed for Q1. A decrease in the CIDNP intensity was observed in the presence of metal ions (Figure 8).

**Figure 9:**
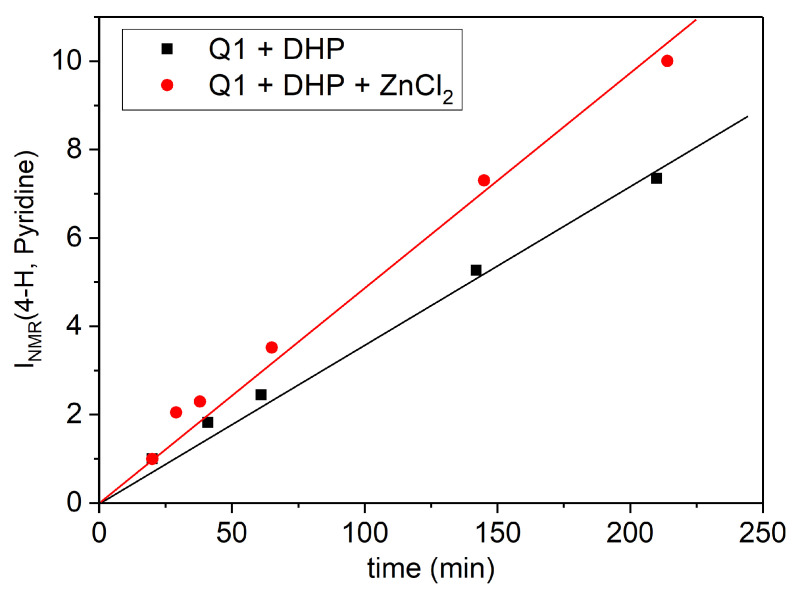
Time dependence of the 4-H pyridine ^1^H NMR signal (in arbitrary units) in the dark reaction between 0.9 mM 2-phenyl-4-(butylamino)naphtho [2,3-h]quinoline-7,12-dione (Q1) and 6 mM 2,6-dimethyl-3,5-dicarbmethoxy-1,4-dihydropyridine (DHP) in the absence (▪) and in the presence (●) of 0.9 mM of ZnCl_2_ in MeOD. Lines corresponds to linear approximation.

Additional studies were undertaken in order to compare the effect of the complex formation in the photochemical reaction with that under dark conditions. Furthermore, because of the direct interference of Cu^2+^/Fe^3+^ in the redox reaction between Q1 and DHP, it was also decided to study the reaction in the presence of Zn^2+^, for which photochemical data were already available [17]. Under these conditions, an increase in the electron transfer (k_e-_) rate between Q1 and DHP was observed based on the 4-H pyridine line in the ^1^H NMR spectra (Figure 9).

## 3. Materials and Methods

The quinone-quinoline chelator, 2-phenyl-4-(butylamino)naphtho [2,3-h] quinoline-7,12-dione (Q1, Figure 1), was synthesized according to the procedure described by Dikalov et al. [74]. Deuterated solvents (CD_3_OD and CD_3_CN, 99.8% D) were obtained from Sigma Aldrich and were used as supplied. 2,6-Dimethyl-3,5-dicarbomethoxy-1,4-dihydropyridine (DHP) was received from Prof. G. Duburs, the Institute of Organic Synthesis, Riga, Latvia. Salts ZnCl_2_, CuSO_4_, and FeBr_3_ 99% were obtained from Sigma Aldrich, Moscow, Russia.

The ^1^H-NMR spectra were recorded using a Bruker AVHD-500 spectrometer (500 MHz, τ(π/2) = 10 μs) (BioSpin, Rheinstetten, Germany). The CIDNP spectra were recorded using a Bruker DPX-200 spectrometer (200 MHz, τ(π/2) = 3.4 μs) (Rheinstetten, Germany). For the CIDNP experiments, all samples in standard 5 mm Pyrex NMR tubes were irradiated directly in the NMR probe of a Bruker DPX200 NMR spectrometer with laser light (EMG 101 MSC Lambda Physik; λ = 308 nm, pulse duration 15 ns, and average pulse energy 100 mJ). The samples were bubbled with argon for 10–15 min to remove dissolved oxygen just before photolysis. Pseudo steady-state photo-CIDNP (PSS) experiments were performed using a standard pulse sequence: presaturation—delay1- pulse τ(π)—delay2 (16 laser flashes with repetition rate 50 Hz during delay 2)—observation pulse τ(π/2)—acquisition. Delay1/delay2 ≈ 1.1 was used to remove the residual signals of the solvents and solutes. After laser irradiation, the ^1^H spectra of the products were recorded on a Bruker Avance HD III NMR spectrometer. The EPR spectra of the samples were recorded on a Bruker ER 200D-SRC X-band EPR spectrometer at the temperature of 25 °C. The instrumental parameters were as follows: microwave power, 20 mW; field modulation, 1G; receiver gain, 8 × 10^5^; and time constant, 200 ms.

## 4. Conclusions

The study of the dark and photo-induced oxidation of 1,4-DHP (synthetic analogue of NADH) by Q1 chelating complexes demonstrated that chelation has a strong effect on the generation of the semiquinone radicals and free radicals of 1,4-DHP. It was demonstrated that the complex formation of this quinone-quinoline chelator with Cu^2+^ and Fe^3+^ led to an increase in free radical production in the case of using DHP as a reducing agent, which was also previously shown for Zn^2+^ and Ca^2+^. At the same time, a decrease in free radical production was observed in the photoinduced reaction of emodin with DHP in the presence of both Cu^2+^ and Fe^3+^. Thus, Q1 complexes with Cu^2+^ and Fe^3+^ appear to be more effective in free radical production than the emodin metal complexes in the model reaction with DHP. Furthermore, it was shown that the semiquinone radical-anion underwent a degenerate electron exchange with the parent Q1, manifesting itself as severe broadening or even the disappearance of the aromatic part of the Q1 ^1^H NMR spectrum. The observed increase in the ^3^Q1 quenching rate by DHP in the case of the Q1 complex with either Cu^2+^ or Fe^3+^ was correlated with an increase in the bimolecular rate constant of the electron transfer between Q1 and DHP in the dark reaction. It can be assumed that these effects were due to the influence of chelation on the electrochemical half-wave potential of Q1, which was also demonstrated and reported in earlier studies in the presence of Zn^2+^ and Ca^2+^. 

Due to the similarity of the enzymatic and photoinduced free radical production by quinones, the mechanism of the influence of chelation on DHP oxidation could be useful for understanding the features of enzymatic NADH oxidation and the anti-tumor activity of quinone-quinoline-based chelators. Further in vitro and in vivo studies of the cytotoxic activity of Q1 and its chelate metal complexes in dark and light conditions have been planned to test the hypothesis of the possible increase in the cytotoxic activity of this quinone-quinoline chelator and its metal complexes. 

In this context, it can be suggested that the results of these studies might be important for the development of new anticancer compounds, active in both dark conditions and in photodynamic therapy.

## Data Availability

Data are contained within the article.

## Abbreviations

CIDNP	chemically induced dynamic nuclear polarization
DHP	2,6-dimethyl-3,5-dicarbmethoxy-1,4-dihydropyridine
EPR	electron paramagnetic resonance
GSH	glutathione
ISC	intersystem crossing
MeOD or CD_3_OD	deuterated methanol
NADH	nicotinamide adenine dinucleotide
NMR	nuclear magnetic resonance
RP	radical pair
Pyr	pyridine product
ROS	reactive oxygen species
Q1	2-phenyl-4-(butylamino)naphtho [2,3-h]quinoline- 7,12-dione
^3^Q1	quinone triplet state
